# T1 mapping of the myocardium: Intra-individual assessment of the effect of field strength, cardiac cycle and variation by myocardial region

**DOI:** 10.1186/1532-429X-14-27

**Published:** 2012-05-01

**Authors:** Nadine Kawel, Marcelo Nacif, Anna Zavodni, Jacquin Jones, Songtao Liu, Christopher T Sibley, David A Bluemke

**Affiliations:** 1 Radiology and Imaging Sciences and Molecular Biomedical Imaging Laboratory, National Institute of Biomedical Imaging and Bioengineering, Bethesda, MD, USA; 2 National Institutes of Health, 10 Center Drive, Bethesda, MD 20892-1074, USA

**Keywords:** T1 mapping, Modified Look-Locker inversion recovery, Extracellular volume fraction, ECV, Field strength

## Abstract

**Background:**

Myocardial T1 relaxation time (T1 time) and extracellular volume fraction (ECV) are altered in the presence of myocardial fibrosis. The purpose of this study was to evaluate acquisition factors that may result in variation of measured T1 time and ECV including magnetic field strength, cardiac phase and myocardial region.

**Methods:**

31 study subjects were enrolled and underwent one cardiovascular MR exam at 1.5 T and two exams at 3 T, each on separate days. A Modified Look-Locker Inversion Recovery (MOLLI) sequence was acquired before and 5, 10, 12, 20, 25 and 30 min after administration of 0.15 mmol/kg gadopentetate dimeglumine (Gd-DTPA; Magnevist) at 1.5 T (*exam 1*). For *exam 2*, MOLLI sequences were acquired at 3 T both during diastole and systole, before and after administration of Gd-DTPA (0.15 mmol/kg Magnevist).*Exam 3* was identical to exam 2 except gadobenate dimeglumine was administered (Gd-BOPTA; 0.1 mmol/kg Multihance). T1 times were measured in myocardium and blood. ECV was calculated by (ΔR1_myocardium_/ΔR1_blood_)*(1-hematocrit)_._

**Results:**

Before gadolinium, T1 times of myocardium and blood were significantly greater at 3 T versus 1.5 T (28% and 31% greater, respectively, p < 0.001); after gadolinium, 3 T values remained greater than those at 1.5 T (14% and 12% greater for myocardium and blood at 3 T with Gd-DTPA, respectively, p < 0.0001 and 18% and 15% greater at 3 T with Gd-BOPTA, respectively, p < 0.0001). However, ECV did not vary significantly with field strength when using the same contrast agent at equimolar dose (p = 0.2). Myocardial T1 time was 1% shorter at systole compared to diastole pre-contrast and 2% shorter at diastole compared to systole post-contrast (p < 0.01). ECV values were greater during diastole compared to systole on average by 0.01 (p < 0.01 to p < 0.0001). ECV was significantly higher for the septum compared to the non-septal myocardium for all three exams (p < 0.0001-0.01) with mean absolute differences of 0.01, 0.004, and 0.07, respectively, for exams 1, 2 and 3.

**Conclusion:**

ECV is similar at field strengths of 1.5 T and 3 T. Due to minor variations in T1 time and ECV during the cardiac cycle and in different myocardial regions, T1 measurements should be obtained at the same cardiac phase and myocardial region in order to obtain consistent results.

## Background

*Focal* ischemic and non-ischemic scar of the myocardium can be reliably detected with late gadolinium enhancement (LGE) magnetic resonance (MR) [1]. Using LGE, the threshold limit of detection of focally damaged myocardium was reported to be 5% per left ventricular slice [2]. A wide variety of non-ischemic cardiomyopathies are associated with *diffuse* myocardial fibrosis that is characterized by an increase in collagen fibers [3,4]. In these conditions, the LGE technique is unlikely to identify subtle alterations in diffuse fibrosis.

An alternative approach to characterize myocardial tissue composition is direct measurement of myocardial T1 relaxation time (T1 time). Alterations in T1 time have been related to variation in myocardial fibrosis: an increase in extracellular volume (associated with higher collagen content) is associated with a greater T1 time on non-contrast MR imaging. After gadolinium administration, T1 times are lower in association with greater collagen content [5]. The modified Look-Locker Inversion Recovery (MOLLI) is a convenient method to measure T1 times during a single breath-hold [6]. The extracellular volume fraction (ECV) characterizes the distribution of a gadolinium contrast agent in the myocardium relative to blood pool. ECV can be approximated from the relaxation rates (R1) of myocardium and blood pool before versus after gadolinium administration, corrected for the blood hematocrit [7-9].

Several technical parameters have the potential to alter T1 time and ECV values. T1 mapping has been performed at both 1.5 T [5,6,10,11] and 3 T [8,12], but intra-individual comparison in a larger sample size has not been performed yet. Myocardial blood volume varies between systole and diastole but the sensitivity of T1 mapping to these variations is also unknown. In addition, T1 time and ECV values may be measured globally or regionally; regional measurement may be affected by local alteration in field homogeneity or differences in motion related artefacts. Thus the purpose of this paper was to evaluate the effect of these parameters (field strength, region by region comparison and cardiac cycle) in order to establish the magnitude of the effect size in relationship to T1 time and ECV.

## Methods

### Study population

31 study participants (20 women; mean age ± SD, 28 ± 6 years; age range, 18–40 years) signed informed consent as part of an ongoing institutional review board approved study. Each participant was asked to undergo three MR scans with different field strength and contrast media, respectively. At each of the 3 separate MR examinations, clinical history and physical examination were assessed to be stable, and laboratory values were repeated. Since “normal aging” is associated with increasing diffuse fibrosis by histology, the upper limit of age to participate in the study was set to 40 years in order to establish a homogenous group of healthy volunteers and reduce the potential for age related differences [13]. All study participants were free of clinically evident illness or elevated cardiovascular risk factors. The ECG obtained prior to the first MR exam did not show an abnormality and the physical exam performed by a physician did not reveal a pathologic finding in any of the participants.

### MR acquisition

Each study subject was scanned three times: once using a 1.5 T MR scanner (Avanto, Siemens Medical Solutions, Erlangen, Germany) (exam 1) and twice using a 3 T MR scanner (Verio, Siemens Medical Solutions, Erlangen, Germany) (exam 2 and 3) using a 32-channel cardiac coil for all exams. Although the MR scans are labeled as exam 1, 2 and 3, the order of the exams was influenced by scanner availability and varied in several subjects.

The MOLLI sequence was acquired in mid-ventricular short axis views pre contrast and 5, 10, 12, 20, 25 and 30 minutes after intravenous contrast administration at the 1.5 T MR scanner (exam 1). The exams at the 3 T scanner were performed at mid- to end-diastole and systole (exam 2 and 3). In order to have more time points for comparison and a longer time span after contrast administration, additional acquisitions of MOLLI were performed at 15, 35, 40 and 45 min. In preliminary tests ideal delay time for image acquisition at systole was evaluated in additional volunteers. A delay time of 150 ms (early systole) after the R-wave resulted consistently in the best image quality and was therefore used for the current study.

For the scans at 1.5 T (*exam 1* ) and one of the scans performed at 3 T (*exam 2*), gadopentetate dimeglumine (Gd-DTPA; Magnevist, Bayer Healthcare) at a dose of 0.15 mmol/kg was administered as a bolus of 2 ml/s followed by a 30 ml saline chaser bolus at the same flow rate using a power injector. In *exam 3*, imaging was also performed at 3 T and a bolus of gadobenate dimeglumine (Gd-BOPTA; Multihance, Bracco Dignostics) was administered at a dose of 0.1 mmol/kg at the same flow rate. A lower dose was chosen in order to compensate for the higher relaxivity and to be consistent with our institution’s clinical routine [14-16]. Scan parameters were as follows: TE/TR 1.14/2.7 ms (1.5 T), 1.03/2.4 ms (3 T); flip angle 35°; bandwidth 977 Hz/Px (1.5 T), 1002 Hz/Px (3 T); minimum TI 125–150 ms; TI increment 80 ms; slice thickness 8 mm; iPAT factor (GRAPPA) 2.

At the time of the first MR study, a complete cardiac MR examination including cine steady state free precession (SSFP) images of the entire heart in short and long axis as well as late gadolinium enhancement (LGE) sequences were obtained in order to exclude occult myocardial disease.

### Image analysis

T1 maps were generated using MRmap [17]. Manual motion correction was performed when necessary. T1 maps were transferred to QMass V.7.2 (Medis Medical Imaging Systems, Netherlands). Left ventricular endocardial and epicardial contours were drawn manually while segments were defined automatically by the software after marking the border between segment 7 and 8. T1 times at the mid-cavity short axis slice of the left ventricle (American heart association [AHA] segments 7–12 [18]) were determined for each segment. An overall T1 time for each slice was also calculated, excluding images with severely impaired image quality; score 4 (see below). T1 time of the blood pool was measured by manually drawing a region of interest in the blood pool of the left ventricular cavity taking care to avoid the papillary muscles. Since a heart rate dependency for long T1 times has been demonstrated, heart rate correction was performed for the pre contrast T1 maps acquired at the 3 T scanner using custom made IDL-based software [19]. For 10 randomly chosen subjects measurements were performed by a second independent reader to assess inter-observer variability. The ECV was calculated as ΔR1_myocardium_/ΔR1_blood_ * (1-hematocrit), where ΔR1_myocardium_ = 1/T1_myocardium pre contrast_ - 1/T1_myocardium post contrast_ and ΔR1_blood_ = 1/T1_blood pre contrast_ - 1/T1_blood post contrast_[7]. For images acquired at systole and diastole each myocardial segment was assigned a quality score ranging from 1 to 4 (1 = perfect image quality without artifacts, 2 = slightly impaired image quality, e.g. by slight blurring, 3 = severely impaired image quality, but not involving the whole segment, 4 = severely impaired image quality involving the whole segment). An overall image score for the entire mid-cavity slice was calculated by the sum of the 6 segments.

For comparison of regional variation of T1 times the mean value of AHA segments 8 and 9 were defined as the septum and the mean value of AHA segments 7, 10, 11 and 12 were defined as non-septal myocardium. The septal and non-septal myocardium were chosen for comparison related to results of preliminary analysis that revealed a difference of T1 times in these two regions.

Left ventricular ejection fraction was obtained using Cardiac Image Modeller (CIM) (Auckland, New Zealand) [20].

### Statistical analysis

Statistical analysis was performed using PASW (version 19) and SAS (version 9.2) statistical software. A p-value <0.05 was considered to be statistically significant.

The continuous variables T1 time (ms) and ECV are expressed as mean ± SD. Myocardial T1 times obtained before contrast administration were compared using a paired *t*-test. A linear mixed-model analysis was performed with the log transformed T1 times for blood and myocardium to compare T1 time among groups over time after contrast administration. Linear mixed-model analysis with the ECV values to identify group related differences was also performed. Both sets of models evaluated group, time and group-by-time interaction by study subjects as random effects.

Quality scores for images acquired at systole and diastole were compared using the Wilcoxon signed rank test.

To compare inter-observer agreement, analysis according to Bland-Altman was performed and Pearson’s correlation coefficient (poor agreement = 0; slight = 0.01-0.20; fair = 0.21-0.40; moderate = 0.41-0.60; good = 0.61-0.80, and excellent = 0.81-1.00 agreement) and the intraclass correlation coefficient (ICC) using a two-way random model (ICC < 0.40 = poor; ICC ≥ 0.40 to 0.75 = fair to good; ICC > 0.75 = excellent agreement) were calculated.

## Results

Of 31 study participants, 28 completed the scan at 1.5 T with Gd-DTPA (exam 1), 29 completed the scan at 3 T with Gd-DTPA (exam 2) and 24 completed the scan at 3 T with Gd-BOPTA (exam 3). 23 volunteers completed all three exams. Average delay between one exam and the following exam was 58 ± 31 days. Of the 8 volunteers who did not complete all scans, 6 volunteers refused to complete the three scans for various reasons, in one subject the scan had to be terminated after contrast injection (Gd-BOPTA) related to nausea and vomiting and one volunteer was pregnant.

Of 1176 segments evaluated on images acquired at 1.5 T, 27 (2.3%) had to be excluded due to severe artifacts (quality score 4). Similar percentages of segments were excluded of the other exams: 39/1914 (2%) (3 T Gd-DTPA, diastole), 27/1914 (1.6%) (3 T Gd-DTPA, systole), 15/1566 (1.0%) (3 T Gd-BOPTA, diastole) and 5/1368 (0.4%) (3 T Gd-BOPTA, systole). In 2 volunteers scanned at 3 T with Gd-BOPTA (exam 3), 5 maps could not be obtained due to a scanner malfunction and in one other volunteer images were acquired at diastole only.

Global and regional left ventricular function was normal in all volunteers (mean ejection fraction ± SD: 60.4 ± 4.5%). Late gadolinium enhancement (LGE) MR images did not show myocardial scar in any of the participants. Table 1 shows further characteristics of the study participants.

**Table 1:** Characteristics of study participants (n = 31)

**Characteristics**	**1.5 T Gd-DTPA** **(exam 1)**	**3 T Gd-DTPA** **(exam 2)**	**3 T Gd-BOPTA** **(exam 3)**
Subjects, n	28	29	24
Age, years	27.6 ± 5.8	28.3 ± 6.1	28.1 ± 5.9
Men, n (%)	10 (36)	9 (31)	8 (33)
Weight in kg, mean ± SD	72 ± 13	72 ± 14	69 ± 13
Hematocrit in %, mean ± SD	41 ± 6	40 ± 4	40 ± 4
Creatinine in mg/dl, mean ± SD	0.7 ± 0.2	0.8 ± 0.2	0.8 ± 0.2
Gadolinium in ml, mean ± SD	21.5 ± 4	21.5 ± 4	13.9 ± 3

Of note, differences of T1 times are shown as absolute difference in milliseconds and/or relative difference in percent. ECV is reported as a ratio. Differences in ECV are mentioned as absolute differences of the ratio and/or the relative difference in percent.

### Intra-individual evaluation of ECV and T1 time at 1.5 T versus 3 T

Comparison of 1.5 T and 3 T was performed for volunteers who completed all three exams (n = 23). As expected, before contrast administration, T1 time of myocardium was 28% higher and T1 time of blood was 31% higher for exams acquired at 3 T compared to 1.5 T (p < 0.0001).

**Figure 1:**
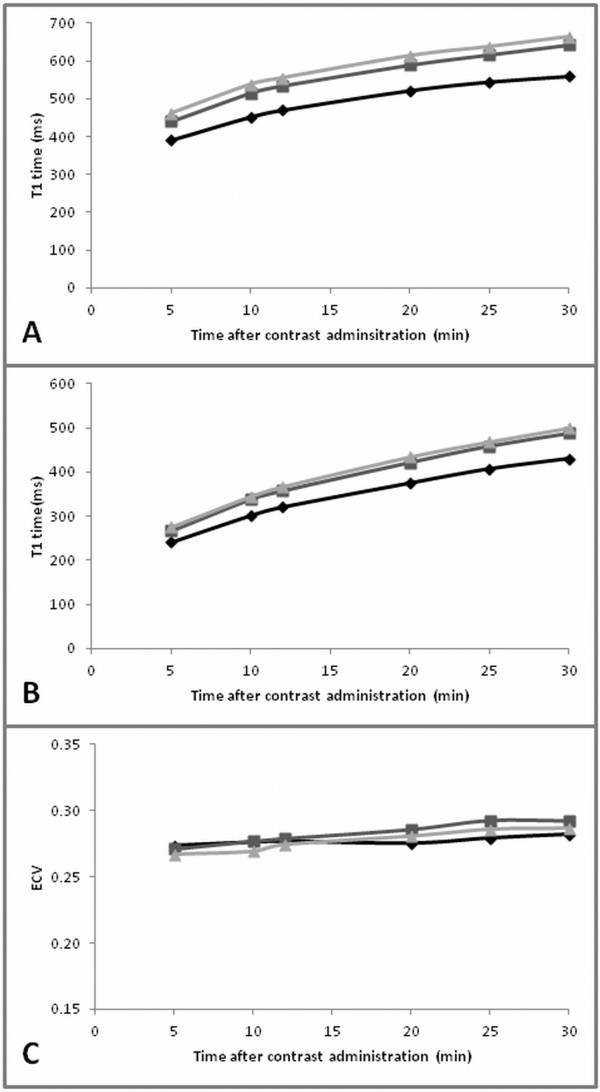
**Intra-individual evaluation of ECV and T1 time at 1.5 T versus 3 T.** Effect of field strength for exam 1 (1.5 T Gd-DTPA; black diamond), exam 2 (3 T Gd-DTPA; dark grey square), and exam 3 (3 T Gd-BOPTA; light grey triangle). (**A**) T1 time of myocardium. (**B**) T1 time of blood. (**C**) ECV over time. Results shown as the average for study subjects who completed all three exams (n = 23).

For the measurements between 5 and 30 minutes after contrast administration, mean myocardial T1 time was on average 67 ms (14%) higher for exam 2 (3 T, Gd-DTPA) compared to exam 1 (1.5 T, Gd-DTPA) and it was 88 ms (18%) higher for exam 3 (3 T, Gd-BOPTA) compared to exam 1 (1.5 T, Gd-DTPA) (p < 0.0001, both comparisons). Results for T1 time of blood were similar: T1 time of blood was on average 43 ms (12%) higher for exam 2 (3 T, Gd-DTPA) compared to exam 1 (1.5 T, Gd-DTPA) and it was 52 ms (15%) higher for exam 3 compared to exam 1 (1.5 T, Gd-DTPA) (p < 0.0001, both comparisons) (Figure 1).

There was no significant difference for ECV between 1.5 T and 3 T using Gd-DTPA (p = 0.2) while there was a significant difference for the comparison between 1.5 T using Gd-DTPA (exam 1) and 3 T using Gd-BOPTA (exam 3) (p = 0.01). ECV obtained at 5, 10 and 12 minutes was on average 0.006 (2.0%) lower for exam 3 (3 T, Gd-BOPTA) compared to exam 1 (1.5 T, Gd-DTPA) while mean ECV obtained at 20, 25 and 30 minutes was 0.005 (2.1%) higher for exam 3 (3 T, Gd-BOPTA) compared to exam 1 (1.5 T, Gd-DTPA) (Figure 1).

Intra-individual variation in ECV was rather large. Including all 24 subjects who completed all three exams, the mean absolute difference between ECV values of all time points was between 0.09 and 0.1.

### Effect of cardiac phase

In exam 2 (3 T, Gd-DTPA) and exam 3 (3 T, Gd-BOPTA) MOLLI was acquired at mid- to end-diastole and early systole. In these subjects, we assessed the variation in T1 time and ECV during diastole compared to systole.

**Figure 2:**
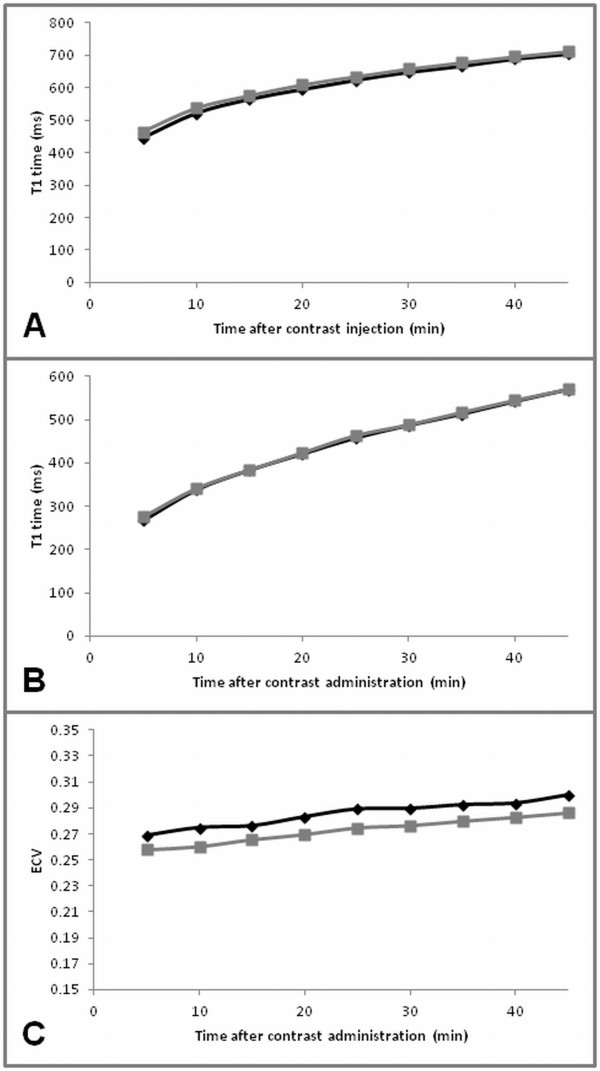
**Effect of cardiac phase.** Diastolic (black diamond) versus systolic (grey square) comparison of T1 time and ECV for the myocardium (**A**) and blood pool (**B**) and ECV (**C**) over time exemplarily for exam 2 (3 T Gd-DTPA). Results for exam 3 (3 T Gd-BOPTA) were similar (not shown).

Prior to contrast administration myocardial T1 time was significantly lower at systole compared to diastole for both exams at 3 T (p < 0.01), consistent with lower myocardial blood volume at systole. However, the mean difference averaged over all time points was only 15 ms (about 1% change in the mean T1 time) for exam 2 and 5 ms (about 0.4%) for exam 3 (Figure 2).

After contrast administration, mean myocardial T1 time was significantly lower at diastole compared to systole (p < 0.01) but mean differences in myocardial T1 time between cardiac phases were small: 12 ms (2%) for Gd-DTPA and 13 ms (2%) for Gd-BOPTA. As expected, blood pool showed no variation with cardiac cycle (p = 0.3 [Gd-DTPA]; p = 0.2 [Gd-BOPTA]). Consequently ECV varied as a function of cardiac cycle; although mean group differences for ECV over repeated measurements were statistically significant (p < 0.0001 [Gd-DTPA]; p < 0.01 [Gd-BOPTA]), the mean ECV was on average only 0.01 higher in diastole compared to systole (Figure 2).

Image quality scores per segment are displayed in Table 2. For the exam with Gd-DTPA, the overall image quality score for the entire slice of 280 T1 maps of images acquired at diastole was 12.4 ± 2.9 and 11.7 ± 3.3 for images acquired in systole (p < 0.0001). For 230 T1 maps of images acquired with Gd-BOPTA the mean image quality was 11.4 ± 2.3 for diastole and 10.1 ± 2.7 for systole (p < 0.0001) (lower score indicated better image quality).

**Table 2:** Image quality score

	**Exam 2 (3 T Gd-DTPA)**		**Exam 3 (3 T Gd-BOPTA)**	
	**diastole**	**systole**	**diastole**	**systole**
**Seg 7 (anterior)**	2.2 ± 0.7	2.2 ± 0.6	2.0 ± 0.6	2.0 ± 0.5
**Seg 8 (anteroseptal)**	1.7 ± 0.6	1.6 ± 0.6	1.7 ± 0.5	1.5 ± 0.6
**Seg 9 (inferoseptal)**	1.6 ± 0.6	1.5 ± 0.6	1.4 ± 0.6	1.3 ± 0.5
**Seg 10 (inferior)**	2.4 ± 0.7	2.1 ± 0.8	2.1 ± 0.6	1.8 ± 0.6
**Seg 11 (inferolateral)**	2.1 ± 0.7	2.2 ± 0.8	2.2 ± 0.6	1.8 ± 0.7
**Seg 12 (interolateral)**	2.2 ± 0.7	2.1 ± 0.8	2.0 ± 0.5	1.7 ± 0.7

Lower image quality score indicates better image quality; Seg = segment.

Regionally, the septal segments 8 and 9 had the best image quality score over all scans and time points. In the majority of scans segment 12 (anterolateral) had the third best quality score. Overall, segment 7 (anterior) and 11 (inferolateral) had the worst quality scores (Table 2).

### Regional variation in T1 time and ECV: Septum versus non-septal myocardium

Before contrast administration T1 times were statistically significantly longer for the septum compared to the non-septal myocardium at 3 T (p < 0.01 [exam 2] and p < 0.0001 [exam 3], respectively) but not at 1.5 T (p = 0.09). However, T1 time at 3 T was on average only 19 ms (1.5%) (exam 2) and 24 ms (1.9%) (exam 3), respectively longer for the septal myocardium.

**Figure 3:**
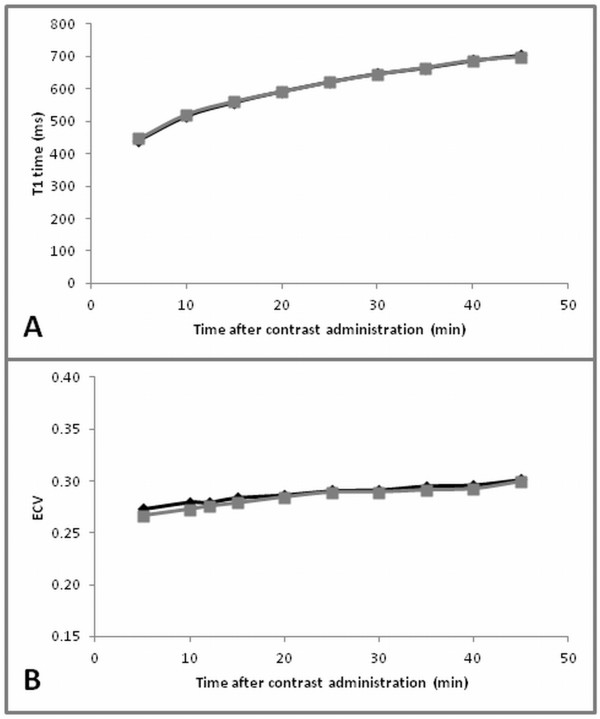
**Regional variation in T1 time and ECV measurement.** Comparison of septal (black diamonds) and non-septal myocardium (grey squares). (**A**) T1 time of myocardium and (**B**) ECV exemplarily for exam 2 (3 T Gd-DTPA). Results for exam 3 (3 T Gd-BOPTA) were similar (not shown).

Mean T1 time after contrast administration differed statistically significantly between the two groups (septum versus non-septal myocardium) for the exams performed at 1.5 T (p = 0.02) but not for the exams at 3 T (p = 0.4 [Gd-DTPA]; p = 0.6 [Gd-BOPTA]). Although differences in T1 time after contrast administration between the septum and the non-septal myocardium were statistically significant at 1.5 T, the mean T1 time of the non-septal myocardium was on average only 9 ms (or about 2%) longer compared to the septal myocardium. Differences tended to be larger for the acquisitions in the first 20 minutes and decreased after 20 minutes (Figure 3).

ECV was also significantly higher for the septal compared to the non-septal myocardium for all three exams: 1.5 T Gd-DTPA (p < 0.001), 3 T Gd-DTPA (p < 0.01), and 3 T Gd-BOPTA (p < 0.0001). Mean absolute differences were: 0.01, 0.004, and 0.07, respectively (Figure 3).

### Inter-observer agreement

Inter-observer agreement was excellent for measurements of T1 time for myocardium and blood at both field strengths, and for measurements obtained at 3 T in systole and diastole, with ICC ranging from 0.984 to 1.0 (Table 3).

**Table 3:** Inter-observer agreement

	**Pearson’s correlation coefficient (r)**	**p**	**Bias**	**95% Limits of agreement**	**ICC**
**1.5 T Gd-DTPA**					
T1 myocardium	1.0	<0.0001	2.3	−15.7 to 20.2	0.999
T1 blood	1.0	<0.0001	0.7	−18.8 to 20.2	1.0
**3 T Gd-DTPA diastole**					
T1 myocardium	1.0	<0.0001	−0.5	−8.5 to 7.4	1.0
T1 blood	1.0	<0.0001	−0.8	−36.7 to 35.1	0.999
**3 T Gd-DTPA systole**					
T1 myocardium	1.0	<0.0001	5.5	−24.0 to 35.0	0.998
T1 blood	1.0	<0.0001	−1.3	−16.4 to 13.7	1.0
**3 T Gd-BOPTA diastole**					
T1 myocardium	1.0	<0.0001	−7.8	−43.2 to 27.6	0.997
T1 blood	1.0	<0.0001	−7.2	−56.3 to 41.9	0.998
**3 T Gd-BOPTA systole**					
T1 myocardium	0.97	<0.0001	−6.4	−80.4 to 67.7	0.984
T1 blood	0.99	<0.0001	−10.9	−135.2 to 113.5	0.993

ICC = intraclass correlation coefficient.

## Discussion

In the current study we demonstrated that ECV does not vary significantly by field strength (1.5 T versus 3 T) when using the same contrast agent at equimolar dose. Myocardial T1 times and ECV vary significantly between systole and diastole and ECV values vary with myocardial region. However, absolute differences were small and might not be clinically relevant or detectable on an individual patient basis.

### Intra-individual evaluation of ECV and T1 time at 1.5 T versus 3 T

 As expected and according to previous publications, T1 times for blood and myocardium varied significantly with field strength [21,22]. Sharma et al. also described a significant difference for T1 time of myocardium but not for blood [8]. The authors explain the difference by a lesser field dependence due to greater free water content and shorter molecular correlation times in blood. This discrepancy compared to the current study and previous publications might be attributed to a relatively low number of study subjects in the study by Sharma et al. (n = 10) receiving two different contrast doses and a different pulse sequence. Sharma et al. also found a statistically significant difference for the partition coefficient for values obtained at 1.5 T and 3 T. The partition coefficient is similar to ECV used in this study, but is not corrected for hematocrit. In the current study ECV did not vary with field strength when the same contrast agent was used at equimolar dose.

 The variation of ECV between Gd-DTPA and Gd-BOPTA can be explained by the higher relaxivity of Gd-BOPTA related to a weak protein binding capacity. In plasma the contrast agent mainly binds to human serum albumin. Since albumin is present in blood, the distribution between blood and myocardium is expected to be different between the two contrast agents whereas pre-contrast values are the same. This should affect the ratio of the change in relaxation rate of myocardium and blood and could explain the difference in ECV values [23].

Of note is the relatively large inter-individual variation in ECV values. The mean absolute inter-individual variation was 0.09 to 0.1. This will hamper establishment of cut-off values between normal and disease and needs to be further evaluated in future studies.

### Effect of cardiac phase

Differences in T1 time between systole and diastole are mainly related to changes in the myocardial blood volume. In the rabbit heart vascular space was measured as 0.15 ± 0.04 ml/g [24]. In the left ventricular free wall of rat hearts Judd et al. demonstrated a decrease in intramyocardial blood volume by 42% from diastole to systole. Blood volume changed from 8.6 ± 1.3 ml/100 g to 5.0 ± 0.7 ml/100 g [25]. Changes from diastole to systole in capillary volumes are larger compared to pre and post capillary microvessels (decrease of 32% versus 48%) and changes in the septum have been shown to be larger compared to the lateral wall (decrease of 25% versus 18.2%) [26,27]. Differences in reported numbers are related to different measurement techniques and study subjects as well as in vivo versus ex vivo studies.

In a recent publication Wansapura et al. evaluated cyclic changes of myocardial blood volume by T1 mapping in seven volunteers without contrast administration using a dual flip angle technique and a fast gradient echo sequence [28]. They describe a decrease in T1 time from diastole to systole of 70% in the septum and 43% in the lateral wall. With a decrease in T1 time from diastole to systole of about 1% prior to contrast administration and an increase from diastole to systole of about 2% after contrast administration, differences in T1 time of the current study are substantially smaller compared to measurements reported by Wansapura et al. Although myocardial blood volume changes significantly from diastole to systole, the blood volume accounts for a small fraction of the entire myocardial volume only and decreases by 3.6 ml/100 g from diastole to systole, assuming that conditions of the human heart are similar to the animal heart [25]. The small change of T1 time from diastole to systole in the current study concurs with the change in myocardial blood volume as a relatively small fraction of the entire myocardial volume.

Since the myocardium is thicker during systole and images yielded a better image quality in the current study, it may be advantageous to measure T1 times at systole. In the current study, absolute variation of myocardial T1 time and ECV values over the cardiac cycle was significant. However, absolute changes were small (<2%) and might not be clinically relevant.

**Figure 4:**
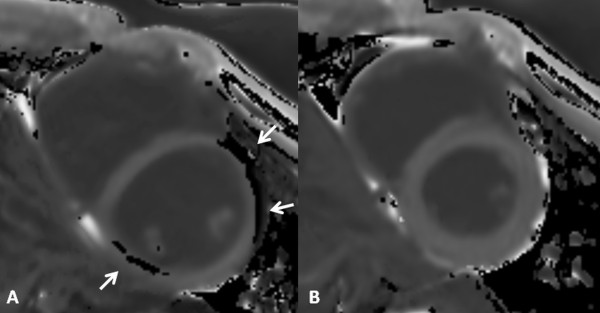
**Modified Look-Locker inversion recovery sequence acquired as short axis at mid-cavity 10 minutes after contrast administration in diastole (A) and systole (B).** Despite manual motion correction, there are severe artifacts (grade 4) of the inferior, anterior, and anterolateral myocardial walls (arrows, black areas in the myocardium) of the T1 map calculated from images acquired at diastole (**A**). Good image quality of the corresponding map at systole (**B**).

During a breath hold of about 15 seconds, variation of heart rate might be substantial and changes of up to 20 beats per minute may occur. This variation in heart rate mainly affects duration of diastole and therefore might cause motion related artifacts. In the current study in contrary to the general opinion and despite the rather long acquisition window of about 150 ms of the MOLLI sequence, images acquired at early systole resulted in a constantly good image quality (Figure 4).

### Regional variation in T1 time and ECV measurement: Septum versus non-septal myocardium

In animal experiments, a high regional variability of the myocardial partition coefficient has been demonstrated [29]. Since the septum shows less motion compared to the remaining myocardium, it has been postulated that T1 times might vary with myocardial region due to less motion related measurement errors. The remaining myocardium is also more prone to susceptibility artifacts, particular at 3 T. Further, regional differences in myocardial perfusion might cause variation in T1 time [30]. Messroghli et al. compared T1 times of the base, mid-cavity and apical level of the left ventricle before contrast administration and did not find regional differences [6]. Statistical analysis for differences between the septum and the non-septal myocardium was not performed. In the current study, ECV values varied between regions for both 1.5 T and 3 T with absolute mean differences ranging from 0.004 to 0.07. Therefore, for comparison measurements should be obtained in the same region or cover the entire myocardium of a slice, respectively.

### Strength and limitations

Acquisitions of the MOLLI sequence at systole were performed after the diastolic acquisition. However, the time delay between both acquisitions was in the order of 20 seconds only and T1 times of blood remained stable during this period. Thus this short delay between sequences, although systematic, is unlikely to be responsible for T1 and ECV differences between systole and diastole.

A strength of the study is the higher number of subjects compared to recent publications and the fact that the same subject was scanned three times in order to obtain intra-individual comparison. Since T1 times and ECV values show a relatively high variation between healthy subjects, a study based on intra-individual comparison assures that the detected differences are not related to inter-individual variation.

## Conclusions

T1 mapping is a valuable MR technique to measure myocardial T1 time and calculate the ECV. When using the same contrast at equimolar dose, ECV does not vary with field strength. MOLLI permits measurement of small changes in myocardial blood volume over the cardiac cycle. Further, minor but statistically significant differences in ECV between myocardial regions were detected. But absolute differences were small and might not be relevant depending on the clinical or research application. However, to avoid addition of small differences, repetitive measurements should be obtained in the same cardiac phase and including the same myocardial region.

## Competing interests

The authors declare they have no competing interests.

## Authors’ contribution

NK: study design, data acquisition, data analysis, data interpretation, manuscript drafting; MN: study desing, data analysis, manuscript revision; AZ: data acquisition, data analysis, data interpretation, manuscript revision; JJ: data acquisition, manuscript revision; SL: study design, data acquisition; CS: study design, manuscript revision; DB principal investigator, study design, data interpretation, manuscript revision. All authors read and approved the final manuscript.
